# Asymptomatic bacteriuria in children with sickle cell anemia at The University of Nigeria teaching hospital, Enugu, South East, Nigeria

**DOI:** 10.1186/1824-7288-37-45

**Published:** 2011-09-19

**Authors:** Bartholomew F Chukwu, Henrietta U Okafor, Anthony N Ikefuna

**Affiliations:** 1 Department of Paediatrics, University of Nigeria Teaching Hospital, Enugu (042), Nigeria

**Keywords:** Asymptomatic bacteriuria, Children, Sickle cell anemia

## Abstract

**Background:**

Urinary tract infection (UTI) is a common cause of childhood morbidity and mortality in the tropics. Children with sickle cell anemia (SCA) may have compromised kidney function arising from repeated vaso-occlusive episodes and recurrent symptomatic or asymptomatic UTI.

**Objectives:**

This study aims at determining the prevalence of asymptomatic bacteriuria and sensitivity pattern in children with homozygous sickle haemoglobin compared to children with normal haemoglobin.

**Methods:**

One hundred children with SCA in stable state and 100 children with normal haemoglobin aged 2-12 years were screened for asymptomatic bacteriuria using midstream urine samples. The samples were incubated aerobically at 37°C for 24 hours within one hour of collection. Children whose urine samples yielded significant bacteriuria (≥10^5^cfu/ml) on two consecutive cultures were regarded as having asymptomatic bacteriuria.

**Results:**

Asymptomatic bacteriuria was noted in 6% of children with SCA and occurred more in females than males (F: M = 5:1) when compared to 2% in children with normal haemoglobin. *Escherichia coli *was the commonest organism isolated (33.3%). All the organisms were resistant to co-trimoxazole and ampicillin while most were sensitive to gentamicin, ceftriaxone and the quinolones.

**Conclusion:**

The risk of asymptomatic bacteriuria is three times more common in children with sickle cell anemia than in children with normal haemoglobin. It is therefore important to screen SCA patients, especially the females for UTI and should be treated according to the sensitivity result of the cultured organisms.

## Introduction

Urinary tract infection (UTI) is a common cause of renal disorder in the tropics[1] and causes significant morbidity[2,3] and mortality[4] in children, especially when it is asymptomatic, not detected and treated promptly.

Sickle cell anemia is common in Nigeria with prevalence values ranging from 2% to 3% of the population [5,6] and is associated with increased frequency and severity of infections especially with encapsulated bacteria such as *Streptococcus pneumoniae *and *Haemophilus influenza *as well as salmonella and *Escherichia coli*. Children with sickle cell anemia have increased susceptibility to develop UTI because of altered blood flow in the renal vasculature which causes papillary necrosis and loss of urinary concentrating and acidifying ability of the nephrons with the consequent formation of abnormally dilute and alkaline urine which favours bacterial proliferation [7]. This predisposes them to recurrent UTI and subsequent renal damage. Studies [8,9] have shown that children with sickle cell anemia are more prone to developing UTI and other bacterial infections than those with normal haemoglobin and may have compromised kidney function from repeated vaso-occlusive episodes and recurrent UTI [10]. These factors tend to hasten their development of chronic kidney disease (CKD). However, this trend can be forestalled if the presence of asymptomatic bacteriuria is detected early and appropriate therapy instituted.

The current study, thus examines asymptomatic bacteriuria in children with sickle cell anemia compared to others with normal haemoglobin. Findings from this study will be useful for making recommendations on measures to curtail the development of UTI among patients with sickle cell anemia and thus reduce the burden and consequent morbidity and mortality arising thereby.

## Subjects and Methods

The study was conducted at the University of Nigeria teaching hospital, Enugu, a referral health facility serving five states in South East of Nigeria. It was a prospective study in which sickle cell anemia (HbSS) children aged two years to 12 years who attended the weekly sickle cell clinic of the University of Nigeria teaching hospital (UNTH), Enugu were screened for UTI over an eight month period (December 2007 to July, 2008). A total of 100 children (57 males and 43 females) were selected consecutively as they presented to the clinic. Another 100 school children of the same age group with normal haemoglobin were also selected from day-care centres, nursery and primary schools in Enugu urban to act as controls. Children who had fever or may have had history suggestive of recurrent UTI or use of antibiotics in the previous two weeks were excluded from the study.

Ethical approval for the study was obtained from the ethical and research committee of UNTH, Enugu while written consent was obtained from parents and care-givers before commencing the study

### Laboratory methods

Spot midstream urine specimens were collected into sterile boric acid bottles and transported in an ice containing box for analyses. Venous blood samples were collected from the controls for determination of their genotype using cellulose acetate electrophoresis (pH = 8.6). Only those with HbAA and satisfied other inclusion requirements were enrolled as controls.

Urinalysis was done using Combur-9 ^® ^test strips. Urine sediments of each child's urine after centrifugation at 2000 rpm for five minutes was examined for red blood cells, leukocytes, casts, crystals and bacteria.

The urine samples were cultured in cystine lactose electrolyte deficient (CLED) and blood agar media within one hour of urine collection by employing the semi-quantitative method as described by Guttmann and Stokes [11]. A well calibrated standard wire loop of internal diameter 3 mm and delivering 0.003 ml of urine per loopful was sterilized over a Bunsen burner flame before immersing in well mixed uncentrifuged urine and then streaked into well dried plates of CLED and blood agar media (which were earlier incubated) as described by Uquarhart and Gould[12]. Cultures were incubated aerobically at 37°C for 24 hours and the colonies counted by using a colony counter. Only samples that yielded pure bacterial growth of 10^5 ^or more colony forming units (cfu) per milliliter were regarded as yielding significant bacteriuria. Counts between 10^4 ^and 10^5 ^were repeated while counts ≤ 10^4^cfu were regarded as negative. Mixed growths (growth of more than one species in a sample) especially growth of normal skin flora, picked up during urine collection were regarded as contaminants and therefore disregarded. Second urine samples were collected from children with significant bacteriuria and those whose second urine samples yielded significant bacteriuria were regarded as having asymptomatic bacteriuria.

Organisms were identified using standard identification techniques [13]. Antibiotic sensitivity disc (Abtek biologicals)^® ^containing ampicillin, co-trimoxazole, gentamicin, nitrofurantoin, colistin, tetracycline, nalidix acid, streptomycin and single discs of Drovid (ofloxacin), Siprosan (ciprofloxacin) and Avicef (ceftriaxone) were used and sensitivity pattern determined by the Stokes's method[14] of comparing the zones of inhibition of the test organism.

### Data analyses

Data was analyzed using the Statistical Package for the Social Sciences (SPSS) version 15.0. Proportions were tested using chi-squared test while means were compared with t-tests. Statistical significance was taken as p < 0.05.

## Results

Out of the 100 urine specimens from the subjects, 6(6%) had significant bacterial growth on two consecutive cultures showing a prevalence of asymptomatic bacteriuria of 6% among children with sickle cell anemia. Five (83.3%) of these were females while one(16.3%) was a male giving female to male ratio of 5:1 which is statistically significant(Fischer's exact, p = 0.04). On the other hand, two of 100 urine specimens from the controls (Both females) yielded significant bacteriuria on two consecutive cultures showing a prevalence of 2%, but there was no significant difference in the prevalence of asymptomatic bacteriuria between the subjects and the controls (Fischer's exact, p = 0.14).

Both Gram positive and Gram negative organisms were isolated from the subjects with *Escherichia coli*, a Gram negative enterobacteria isolated from two (33.3%) of the six subjects with asymptomatic bacteriuria while proteus spp, *Staphylococcus albus, Streptococcus faecalis, Staphylococcus aureus *constituted 16.7% each. Staphylococcus aureus and Streptococcus faecalis constituted 50% each of the isolates from the controls. Table 1 shows the distribution and Gram stain of isolated organisms from subjects and controls.

**Table 1:** Frequency distribution and Gram stain reaction of isolated organisms in subjects and controls.

Organism	Gram stain	Subjects	Controls
E.coli	Negative	2(33.3%)	0
Staph albus	Positive	1(16.7%)	1(50%)
Staph aureus	Positive	1(16.7%)	0
Proteus spp	Negative	1(16.6%)	0
Strept faecalis	Positive	1(16.7%)	1(50%)
Total		6(100%)	2(100%)

Sensitivity pattern of the isolated organisms from the subjects showed that 4(66.7%) were sensitive to gentamicin and ciprofloxacin, 3(50%) were sensitive to nitrofurantoin, nalidixic acid and colistin sulphate while 6(100%) were sensitive to ofloxacin and ceftriaxone. All were resistant to co-trimoxazole, ampicillin, tetracycline and streptomycin. Among the controls, the two (100%) isolated organisms were sensitive to gentamicin, ofloxacin and nitrofurantoin while the two (100%) were resistant to co-trimoxazole, ampicillin and streptomycin. Tables 2 and 3 depict the sensitivity of the isolated organisms in subjects and controls respectively.

**Table 2:** Positive culture, organisms and sensitivity pattern in subjects.

S/N	Sex	Organism	Sensitivity
20	F	E.coli	S/G, NT, NA, CL, OF, CP
			R/AM, CT, ST, TET
44	F	Staph albus	S/CF, OF, CP
			R/G, NT, NA, AM, CT, CL, ST, TET
63	M	Staph aureus	S/G, NT, NA, CF, OF
			R/CT, CL, ST, TET, AM, CP
72	F	E.coli	S/CF, CL, NT, OF
			R/G, NA, CP, AM, CT, ST, TET
82	F	Proteus	S/G, NA, CF, CP, OF, CL
			R/NT, AM, CT, ST, TET
88	F	Strept faecalis	S/G, CF, CP, OF
			R/NA, NT, CT, CL, AM, ST, TET

S = Sensitive, R = Resistant

G = Gentamicin, NA = Nalidixic acid, CL = colistin sulphate, OF = Ofloxacin

AM = Ampicillin, TET = Tetracycline, CF = Ceftriaxone, ST = Streptomycin

CT = Cotrimoxazole, NT = Nitrofurantoin, CP = Ciprofloxacine

S/N = Serial number

**Table 3:** Positive culture, organisms and sensitivity pattern in controls.

S/N	Sex	Organism	Sensitivity
30	F	Strept faecalis	S/G, NT, CF, CP, OF
			R/NA, CT, CL, TET, AM, ST
60	F	Staph aureus	S/ST, CL, CT, CP, CF, NA, AM

S = Sensitive, R = Resistant, AM = Ampicillin

G = Gentamicine, NT = Nitrofurantoin, CF = Ceftriaxone, CP = Ciprofloxacine

NA = Nalidixic acid, CT = Cotrimoxazole, CL = Colistin sulphate

TET = Tetracycline, ST = Streptomycin, Of = Ofloxacine

S/N = Serial number.

All the urine samples from subjects and controls with asymptomatic bacteriuria were acidic and none was positive for nitrite test. There was no correlation between pyuria (≥ 5 leukocytes/mm^3 ^of centrifuged urine) and significant bacteriuria.

## Discussion

Studies on asymptomatic bacteriuria in children with sickle cell anaemia are very scanty. However, in this study, the prevalence of asymptomatic bacteriuria in children with sickle cell anemia is 6% and is comparable with the result obtained from Lagos by Ajasin et al [15] who documented 5.8%. This is also comparable to 5.3% documented by Vanessa et al [16] in adult sickle cell patients in Kingston, Jamaica. The proportion of children with sickle cell anemia who had significant bacteriuria in the current study is quite high when compared with the prevalence (2%) among children with normal haemoglobin genotype. At same time it is higher than the figures obtained by Abdulrahman [17] in Kaduna, North West, Nigeria and Okafor [18] in Enugu, South East, Nigeria among children with normal haemoglobin with prevalence of 1% and 2.1% respectively. The current study further supports the greater susceptibility of children with sickle cell anemia to urinary tract infection (UTI) with a threefold increase in asymptomatic bacteriuria when compared to their normal haemoglobin genotype controls. This higher risk is due to the defect in urine concentrating and acidifying abilities of the kidneys of children with sickle cell anemia. This produces abnormally dilute urine which favours bacterial proliferation.

Of the children with sickle cell anemia who had significant bacteriuria in the current study, there were more females than males in a ratio of 5:1. This ratio agrees with that obtained by Tarry et al [19] and Ajasin et al [15] who documented ratios of 10:1 and 3:2 respectively though there were larger sample sizes in the latter studies. This higher risk in the females has been attributed to short course of the female urethra and its proximity to the anal region.

*Escherichia coli*, a Gram negative enterobacteria was isolated from two (33.3%) of the six subjects with asymptomatic bacteriuria while proteus spp, *Staphylococcus albus, Streptococcus faecalis, Staphylococcus aureus *constituted 16.7% each. Previous studies [9,20-23] of asymptomatic bacteriuria both in HbSS and HbAA subjects have identified Gram negative organisms particularly *Escherichia coli *and Klebsiella species as the most prevalent pathogens causing UTI in children. The Gram negative organisms have also been implicated as the most common cause of symptomatic UTI both in children with sickle cell anemia[16,24] and those with normal haemoglobin[21]. The range of pathogens in the current study is similar to that reported by earlier workers [15,19,23] except that Klebsiella species, the second most commonly reported pathogen causing UTI was not isolated in the current study. Thus organisms like *E.coli*, Klebsiella species, Proteus species, *Streptococcus faecalis *and *Staphylococcus aureus *are frequently isolated in subjects with asymptomatic bacteriuria irrespective of haemoglobin genotype. In developed countries of the world, *E. coli *is responsible for 80-90% of all organisms isolated from the urinary tract of children with UTI. The frequency with which this organism causes UTI in the developing countries including Nigeria is low as organisms such as *Staphylococcus aureus, Streptococcus faecalis *and proteus species have larger representation of causative agents in UTI in these less developed countries, probably due to poor environmental and personal hygiene in these less developed countries. It also seems that sickle cell anemia has some effect in the pattern of distribution of the organisms responsible for UTI allowing a greater representation of some other organisms such as Proteus and staphylococcus species. This may be due to the general impairment of the immune system in patients with sickle cell anaemia. The organisms isolated from the two controls with positive culture were both Gram positive organisms. This is at variance with what was obtained by Okafor et al [23] in which Gram negative organisms accounted for 59% of the 17 cases of asymptomatic bacteriuria among pre-school children. This variance may be due to the smaller number of children enrolled as control in the current study.

The sensitivity of the isolated organisms indicates that most of the organisms were resistant to the older antibiotics such as cotrimoxazole, ampicillin, streptomycin and tetracycline (contraindicated in children less than 8 years) both in subjects and controls. This high resistance to the older antibiotics was also noted by other workers both within and outside the country [15,23-25]. The reason for this high resistance may be due to self medication and/or sub-therapeutic (drug pressure) prescription by some health workers as well as poor drug compliance by some patients. It may also be due to intrinsic drug resistance developed by the pathogens.

Urinalysis in both subjects and controls indicated that the urine samples were acidic in all the children with asymptomatic bacteruria. It has been stated that children with sickle cell anaemia have urine acidifying defect but this has not been so in the current study. It may be that the kidneys of these subjects still retain their ability to acidify urine. Nitrite test was negative in all the children with asymptomatic bacteriuria and this may be due to the low sensitivity of this test in detecting bacteriuria as has been observed in a study Wammanda and coworkers [26].

The urine microscopy among the sickle cell anaemia patients showed pyuria ranging from 1-6/hpf but only one with significant bacteriuria had significant pyuria. The sensitivity and specificity of significant pyuria as a determinant of significant bacteriuria in centrifuged urine sample is 61% and 43% respectively [27]. This relatively low sensitivity may explain the presence of significant pyuria in only one of the subjects with asymptomatic bacteriuria. Pyuria is an indication of active inflammation and in cases of asymptomatic bacteriuria as in the current study; significant pyuria may not be detected.

In view of the increased incidence of asymptomatic bacteriuria in children with sickle cell aaemia, it is therefore necessary to screen them for UTI in the clinics using at least the fast and economical tests for detecting bacteriuria such as nitrite and leukocyte esterase tests and that ciprofloxacin may be considered in the empirical treatment of UTI as this has been found to be safe in children [28].

## Competing interests

The authors declare that they have no competing interests.

## Authors' contributions

BF conceived the study, participated in the design and coordination of the study, collected the samples, did the laboratory work, analyzed the result and wrote the final manuscript. HU and AN participated in the design and coordination of the study.

All authors read and approved the final manuscript.
